# HIV/STIs risks between migrant MSM and local MSM: a cross-sectional comparison study in China

**DOI:** 10.7717/peerj.2169

**Published:** 2016-07-13

**Authors:** Jie Wu, Hong Wu, Pengsheng Li, Ciyong Lu

**Affiliations:** Department of Medical Statistics and Epidemiology, Sun Yat-Sen University, Guangzhou, China

**Keywords:** HIV/STIs risk, Migration, MSM, Comparison

## Abstract

**Background.** Internal migration plays a significant role in China’s HIV epidemic. However, few studies have directly compared migrant men who have sex with men (MSM) with local MSM with regard to HIV/sexually transmitted infections (STIs) risks.

**Methods.** We conducted a study in Guangzhou, China, with the aim of understanding the differences in HIV/STIs risks between migrant MSM and local MSM. A cross-sectional study was conducted among 273 migrant MSM and 249 local MSM in Guangzhou, China. Their behavioral and serologic data on HIV/syphilis were collected and compared between the two groups. A multivariate logistic regression was used to estimate the associations between HIV/STIs risks and migratory status.

**Results.** Migrant MSM, compared to local MSM, have higher odds of reporting unprotected anal intercourse (UAI) (*OR* = 1.4; 95% CI [0.9–2.0]) and having multiple homosexual partners (*OR* = 1.2; 95% CI [0.8–1.8]). A lower rate of condom use at homosexual debut was reported in migrant MSM than in local MSM (*OR* = 0.7; 95% CI [0.5–0.9]). Migrant MSM have less odds of reporting HIV/STIs testing in the previous 6 months relative to local MSM (*OR* = 0.5; 95% CI [0.4–0.8]). In addition, migrant MSM demonstrated a lower level of HIV knowledge than local MSM (*OR* = 0.4; 95% CI [0.2–0.8]).

**Conclusion.** Migrant MSM are more likely to engage in sexual risk behaviors, report a lower level of HIV knowledge and have less access to HIV/STIs testing. Further comprehensive interventions targeting migrant MSM are urged.

## Introduction

It was estimated that China has over 245 million internal migrants, accounting for 20% of its total population (2012). A large body of studies has suggested that internal migration plays a significant role in spreading HIV in China (Li et al., 2004; Li et al., 2007; Mantell et al., 2011; Mi et al., 2016; Wang et al., 2010; Yang, Derlega & Luo, 2007). Furthermore, epidemiologic evidence has pointed to that migrants are more likely to engage in HIV/STIs risks, as compared with local residents (Hu et al., 2006; Shi et al., 2009; Yang, Derlega & Luo, 2007). Meanwhile, men who have sex with men (MSM) play an increasingly important role in China’s HIV/AIDS epidemic. According to a recent national report, homosexual transmission of HIV accounted for 21.4% of newly identified HIV/AIDS cases in 2013, increasing from 2.5% in 2006 (2014). Moreover, the existing literature suggests that most MSM in China are migrants, with a proportion ranging from 55% to 88% (Berg et al., 2011; Fan et al., 2012; Feng et al., 2009; Ruan et al., 2009) and approximately 6% of male migrants are MSM (Chen et al., 2015). However, the studies that compared migrant MSM and local MSM with respect to HIV/STIs risk profile were generally limited despite that several studies have been conducted to understand HIV/STIs risks in the migrant MSM population.

In the past decade, a growing body of research has been conducted among migrant MSM in China. For example, Mao et al. (2014) reported that an incidence rate of 7.83 per 100 person years for HIV infection and 11.11 per 100 person years for syphilis in a cohort of 511 migrant MSM in Beijing. Several other studies in China have been conducted to understand the HIV testing behaviors (Song et al., 2011), HIV/syphilis prevalence (Wang et al., 2013), bisexual behaviors (Guo et al., 2012), and same-sex disclosure (Guo et al., 2014) in migrant MSM. Two additional studies have been dedicated to exploring HIV/STIs risks in migrant “money boys” (also known as male sex workers) (Lau et al., 2009; Wong et al., 2008). All of these previous researches have contributed to our understanding of HIV/STIs risks among migrant MSM in China. Yet, the inclusion of exclusive migrant MSM did not add our knowledge on the relative HIV/STIs risks of migrant MSM to their local counterparts. Indeed, there were some other studies that recruit both migrant MSM and local MSM suggesting migrant MSM are more likely to be infected with HIV/STIs (He et al., 2006; Ruan et al., 2009; Wu et al., 2013) or engage in unprotected anal intercourse (UAI) (Ruan et al., 2008). However, most of them mixed migrant MSM and local MSM in data analysis and only reported a migratory status-stratified analysis of primary outcome (e.g., HIV infection or UAI). The comprehensive differential contours of HIV/STIs risks between migrant MSM and local MSM are still largely unknown.

So far, few studies have been conducted to directly compare the HIV/STIs risks between migrant MSM and local MSM. Direct comparison may confer additional knowledge on the relation between migration and HIV/STIs risks in the MSM community, and provide information on tailored intervention development. To fill this gap in the literature, we conducted a study in Guangzhou, China, with the aim of understanding the differences of HIV/STIs risks between migrant MSM and local MSM.

## Materials and Methods

### Participants and procedures

We conducted a cross-sectional study between March and August of 2010 in Guangzhou, China. The recruitment method and study procedure have been described in details elsewhere (Jie et al., 2012). Briefly, a convenience sampling was used to select the sample. Trained outreach workers from the MSM community randomly approached potential participants in target MSM venues in Guangzhou (such as bars, parks) and invited them to participate in this study. In addition, we also placed recruitment advertisements on the website of the Lingnan Partner Support Center (LPSC), a local Non-Government Organization (NGO) aiming to prevent the spread of HIV among MSM community in Guangzhou. Participants were screened via telephone by trained study staff and were deemed eligible if they were: (1) male, (2) aged 18 years or older, and (3) reported having oral or anal sex with men in the previous six months. However, our study did not intend to recruit those who stayed in Guangzhou for less than one month in order to exclude temporary visitors or tourists. Participants were compensated approximately $20 for their time.

**Figure 1:**
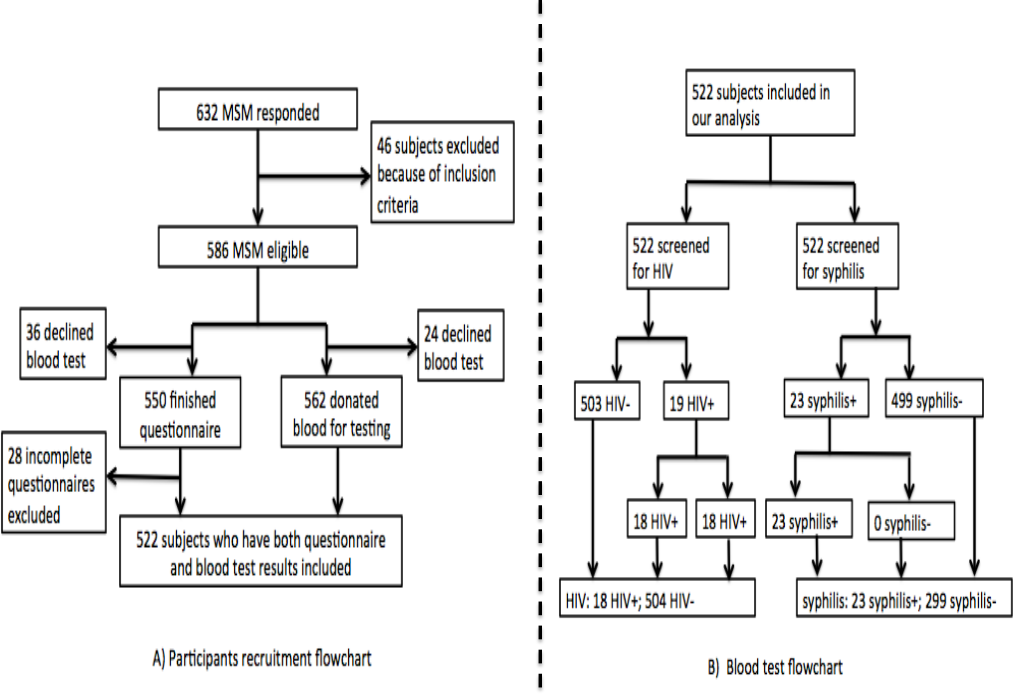
Flowchart of participants recuruitment and blood test.

The study was performed between March and August of 2010. A total of 522 participants completed a self-administered questionnaire and provided blood samples for HIV and syphilis testing. Figure 1 provides a flowchart of participant recruitment and blood test. All the study activities took place in the LPSC.

### Ethical approval

The study protocol was reviewed and approved by the ethics committees of Sun Yat-sen University (SYS IRB#M08-10-03A). Written informed consent was obtained from each participant.

### Measures

We collected the respondents’ migratory status, demographics, sexual risk behaviors, illicit drug use, HIV/STIs testing behaviors, HIV/STIs services utilization and HIV knowledge using a structured questionnaire. The development of our questionnaire was based on a series of standard instruments and scales that have been validated in the previous studies. After finishing the draft of questionnaire, the questionnaire was firstly piloted for its validity and stability. We also asked local NGO staff to review our questionnaire to ensure its adoptability to local MSM community.

#### Migratory status

In our study, we determined the respondents’ migratory status according to their self-reported official household registrations, also known as “Hukou.” For instance, if the subject reported a non-Guangzhou registered “Hukou,” he was defined as a migrant; otherwise he was regarded as a local resident. This measure has been widely used in the previous migration studies in HIV research (Berg et al., 2011; Li et al., 2004; Mao et al., 2014; Ruan et al., 2009; Song et al., 2011). Under the “Hukou” system, each Chinese resident is assigned to a particular place of residence, which usually is the place where they were born. Without having a permanent household registration in the host cities, the migrants cannot access the social welfare benefits available to local residents, such as subsided housing, free education, long-term employment contracts and health care (Solinger, 1999; Zhang, 2001).

#### Socio-demographics and background characteristics

Participants were asked to report their age (continuous and categorized as 18–25 years old, 26–35 years old and 35 years old and above), ethnicity (Han VS. others), educational attainment (secondary school or less, high school and college or above), marital status (unmarried, married and divorced/widowed), monthly income and self-reported sexual orientation (gay, bisexual, heterosexual or uncertain). The other variables collected are described in details in Table 1.

**Table 1:** Socio-demographic characteristics of men who have sex with men (MSM), Guangzhou, China, 2010 (*N* = 522).

Demographics	Overall		Migrant MSM		Local MSM		*p* value
	*N*	%	*N*	%	*N*	%	
Participants	522		273		249		
Age, mean ± SD	29.7 ± 6.6		29.9 ± 6.6		29.5 ± 6.6		0.445
Marital status							0.09
Single	440	84.3	221	81.0	219	88.0	
Married	68	13.0	43	15.7	25	10.0	
Divorced/widowed	14	2.7	9	3.3	5	2.0	
Education							<0.001
Below college	123	23.6	84	15.7	39	30.8	
College and above	399	76.4	189	84.3	210	69.2	
Monthly income^a^							0.001
1,000 and less	87	16.7	31	11.4	56	22.5	
1,001–3,000	309	59.3	178	65.4	131	52.6	
Above 3,000	125	24.0	63	23.2	62	24.9	
Self-reported sexual orientation							0.001
Homosexual or bisexual	378	72.4	181	66.3	197	79.1	
Heterosexual or uncertain	144	27.6	92	33.7	52	20.9	
Common venues for finding partners							0.541
Bar-based venues	17	3.3	9	3.3	8	3.2	
Salon-based venues	29	5.6	12	4.4	17	6.8	
Public facility-based venues	4	0.8	2	0.7	2	0.8	
Internet	441	84.5	237	86.8	204	81.9	
Others	31	5.9	13	4.8	18	7.2	
Satisfaction with current living environment							<0.001
No	258	49.4	157	57.5	101	40.6	
Yes	264	50.6	116	42.5	148	59.4	

**Notes.**

a Missing =1.

Abbreviation SD Standard Deviation MSM Men who have sex with men

The HIV/STIs risk information mainly comprised of two parts including (1) sexual risk behaviors and (2) illicit drug use.

#### Sexual risk behaviors

Participants were asked to recall their homosexual debut and sexual activities in the past 6 months. Information, collected in this section, included age of homosexual debut, condom use at homosexual debut, the number of same-sex sexual partners and condom use with different partners in the past 6 months. In addition, we asked the participants to indicate whether they purchased commercial sex in the past 6 months and condom use in the context of commercial sex. Heterosexual behaviors and condom use in the past 6 months were also collected. The category of multiple homosexual partners was coded positive for individuals who reported more than one receptive or insertive homosexual partner in the last 6 months. Frequency of condom use with a regular, casual and commercial sex partners was indicated as “never,” “sometimes,” “most of time,” or “every time.” In our study, we defined UAI as not using condom every time during anal intercourse with any homosexual partner in the past 6 months.

#### Illicit drug use

Illicit drug use was ascertained by posing a question that asked the participants to indicate whether they used a list of illicit drugs, including marijuana, cocaine, heroin, hallucinogens, amphetamines, methamphetamine, MDMA (“ecstasy”) and ketamine in the past 6 months.

#### HIV/STIs testing behaviors and HIV/STIs services utilization

HIV/STIs testing behaviors were measured by several questions addressing the subjects whether received a testing for HIV or other STIs in the past 6 months. HIV/STIs services utilization was ascertained by summing individual responses to utilization of five HIV/STIs prevention services in the past 6 months, including free condom, free lubricant, counseling, peer education and free reading materials. Participants who have accessed at least one of the five HIV/STIs prevention services were deemed as participants who utilized HIV/STIs services.

#### HIV knowledge

We used an 8-item scale to assess HIV knowledge of participants. The knowledge scale incorporated 8 questions such as “Having dinner with HIV-infected person can transmit HIV,” “Mosquito bites can spread HIV,” etc., all of which required true/false, likely/unlikely, or uncertain responding. This instrument was adopted from a previous study in China (Choi et al., 2007), with a reliable internal consistency (*α* = 0.87). A composite score was retained by summing up all correct answers, with higher score reflecting increased knowledge about the transmission of HIV/AIDS. In addition, we defined that participants obtained a composite score ≥6 were well informed of HIV knowledge.

#### Biological testing for HIV and syphilis

We screened participants for antibodies to HIV (HIV1.2.0, Murex antiHIV, Abbott Laboratories, Abbott Park, IL) and syphilis (ICE Syphilis Murex, Abbott Laboratories, Abbott Park, IL) in the Yuexiu District Center for Disease Control and Prevention Center (CDC) HIV laboratory, Guangzhou City. HIV-positive samples were confirmed using Western Blot (WB; Singapore MP Biomedical Asia Pacific Ltd) and syphilis-positive samples were confirmed by a treponemal antibody test (Serodia Treponema pallidum particle agglutination test; Fujirebio, Tokyo, Japan) in Guangdong CDC HIV confirmatory laboratory. We determined the HIV and syphilis infection status based on the results confirmatory tests that were sent back to us.

### Statistical analysis

Descriptive analysis was performed to compare socio-demographic characteristics, sexual risk behaviors, illicit drug use, HIV/STIs testing, HIV/STIs prevention services utilization, HIV knowledge and HIV/syphilis infection between migrant MSM and local MSM. Chi-square analyses, Fisher exact tests, or *t* tests, whenever applicable, were used to test for the differences between migrant MSM and local MSM. For significant differences, difference of proportion or difference of mean were estimated with 95% Confidence Interval (CI). Because age may modify the prevalence of UAI and HIV/syphilis infection, we further estimated the age-and migratory status-specific prevalence of UAI and HIV/syphilis infection. A multivariate logistic regression model was used to estimate the associations between HIV/STIs risks and migratory status, using local MSM as the reference group. All statistical tests were 2-tailed, and probability values <0.05 were considered statistically significant. STATA, version 12.1, software (StataCorp LP, College Station, Texas) was used for all statistical analysis.

## Results

### Socio-demographic characteristics

A total of 522 MSM, including 273 migrant MSM and 249 local MSM were recruited into our study. Socio-demographic characteristics of participants are presented in Table 1. The mean age of participants was 29.7 years old (SD = 6.6), and migrant MSM and local MSM were comparable with regard to age. Marital status was also similar across the two groups with 84.3% of all participants reporting unmarried. Approximately three-quarters (76.4%) of participants have obtained at least a college degree, and the educational attainment was significantly higher for migrant MSM than local MSM (chi-square test *p* < 0.001, difference of proportions: 15.1%, 95% CI [8.0–22.2%]). In addition, migrant MSM were also less likely to report the lowest income category, compared with migrant MSM. With respective to self-reported sexual orientation, a smaller proportion of migrant MSM self-identified as homosexual or bisexual than local MSM (chi-square test *p* = 0.001, difference of proportions: –12.8%, 95% CI [–20.4–−5.3%]). More details are provided in Table 1.

### Sexual risk behaviors

**Figure 2:**
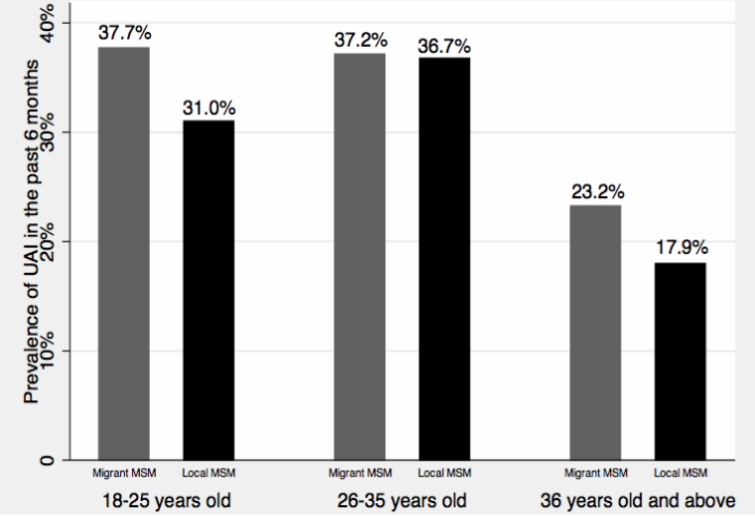
Age-and migratory status-specific prevalence of UAI.

Sexual risk characteristics of participants are summarized in Table 2. Migrant MSM were less likely to report condom use at homosexual debut, compared with local MSM (chi-square test *p* = 0.017, difference of proportions: –10.5%, 95% CI [–19–−1.9%]). Overall, the prevalence of UAI in the previous 6 months was 33.3%, and no significant difference of UAI was found between migrant and local MSM. Figure 2 provides age-and migratory status-specific prevalence of UAI. In general, older MSM were less likely to report UAI than younger MSM, and migrant MSM were more likely to report UAI relative to local MSM, although these differences were not statistically significant. Fifty-five percent of participants had multiple homosexual partners in the previous 6 months, but there was no significant difference of having multiple homosexual partners between migrant MSM and local MSM. Migrant MSM and local MSM were also similar with regard to commercial sex in the past 6 months and condom use in commercial sex. Compared with their local counterparts (10.8%), a larger proportion of migrant MSM reported having heterosexual sex in the previous 6 months (chi-square test *p* = 0.009, difference of proportions: 8.2%, 95% CI [2.2–14.3%]). However, there was no difference in terms of condom use in heterosexual sex in the past 6 months.

**Table 2:** HIV/STIs risks of Men who have sex with men, Guangzhou, China, 2010 (*N* = 522).

Characteristics	Overall		Migrant MSM		Local MSM		*p* value
	*N*	%	*N*	%	*N*	%	
Participants	522		273		249		
Condom use at first homosexual anal sex^*^							0.017
No	255	48.9	147	53.8	108	43.3	
Yes	267	51.1	126	46.2	141	56.6	
UAI in the previous 6 months^b^							0.577
No	348	66.7	179	65.6	169	67.9	
Yes	174	33.3	94	34.4	80	32.1	
Condom use at last homosexual anal sex							0.077
No	177	33.9	83	30.4	94	37.8	
Yes	345	66.1	190	69.6	155	62.2	
Multiple homosexual partners in the past 6 months^a^							0.103
No	238	45.5	115	42.1	123	49.4	
Yes	283	54.5	157	57.9	126	50.6	
Purchasing sex in the previous 6 months							0.938
No	488	93.5	255	93.4	233	93.6	
Yes	34	6.5	18	6.6	16	6.4	
Consistent condom use with commercial sex partners in the previous 6 months							0.744
No	18	52.9	9	50.0	9	56.3	
Yes	16	47.1	9	50.0	7	43.7	
Heterosexual vaginal sex in the previous 6 months^*^							0.009
No	443	84.9	221	81.0	222	89.2	
Yes	79	15.1	52	19.0	27	10.8	
Consistent condom use with female sex partners in the previous 6 months							1.000
No	68	86.1	45	86.5	23	85.2	
Yes	11	13.9	7	13.5	4	14.8	
Illicit drug use							0.824
No	502	96.2	262	96.0	240	96.4	
Yes	20	3.8	11	4.0	9	3.6	
HIV testing in the previous 6 months^*^							0.020
No	298	57.1	169	61.9	129	51.0	
Yes	224	42.9	104	38.1	120	48.2	
STIs testing in the previous 6 months (excluding HIV testing)							0.057
No	324	62.1	180	65.9	144	57.8	
Yes	198	37.9	93	34.1	105	42.2	
Utilization of HIV/STIs prevention services in the previous 6 months							0.715
No	195	37.4	104	38.1	91	36.5
Yes	327	62.6	169	61.9	158	63.5	
HIV knowledge level^*^							0.002
Not-informed	43	8.2	32	11.7	11	4.4	
Informed	479	91.8	241	88.3	238	95.6	
HIV infection							0.239
HIV-negative	504	96.6	261	95.6	243	97.6	
HIV-positive	18	3.4	12	4.4	6	2.4	
Syphilis infection							0.523
Syphilis-negative	499	95.6	259	94.9	240	96.4	
Syphilis-positive	23	4.4	14	5.1	9	3.6	

**Notes.**

a Missing =1.

b UAI with males only.

* *p* < 0.05.

Abbreviation MSM Men who have sex with men UAI Unprotected anal intercourse STIs Sexually transmitted infections

### Other HIV/STIs-related risks and HIV/syphilis infection

**Figure 3:**
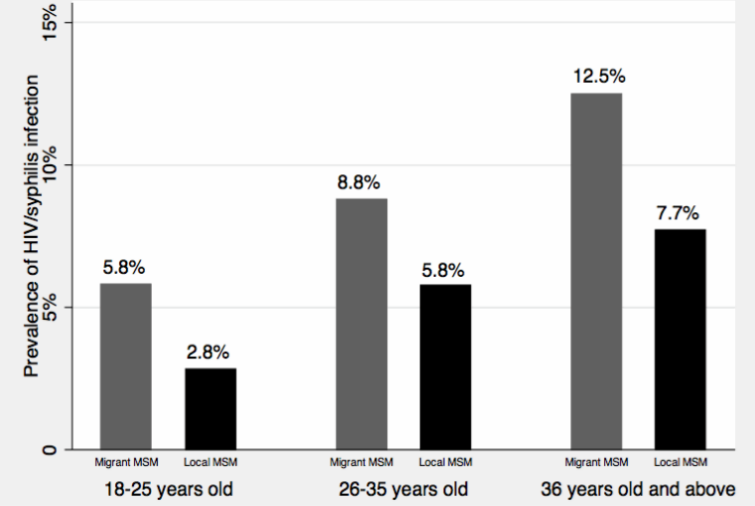
Age-and migratory status-specific prevalence of HIV/syphilis infection.

Table 2 also presents the information on other HIV/STIs-related risks of participants. Overall, the prevalence of illicit drug use in our study was low (3.8%), and no significant difference was identified between the two groups. HIV testing in the previous 6 months was less prevalent in migrant MSM than local MSM (chi-square test *p* = 0.02, difference of proportions: –9.6%, 95% CI [–16.5–−2.6%]). Less than 40% of participants had STIs testing (excluding HIV testing) in the previous 6 months, and no significant difference of having STIs testing was found between the two groups. Similar level of utilization of HIV/STIs prevention services was also reported in both groups. However, migrant MSM had a lower level of HIV knowledge than local MSM (chi-square test *p* = 0.002, difference of proportions: −7.3%, 95% CI [–11.9–−2.7%]). The prevalence of seropositive for HIV and syphilis was 3.4% and 4.4%, respectively, and no significant difference of HIV or syphilis infection was found between migrant MSM and non-migrant MSM. In addition, we combined HIV and syphilis infection data and presented age- and migratory status-specific prevalence of HIV/syphilis infection with Fig. 3. The prevalence of HIV/syphilis infection increased along with the age categories, and the prevalence was higher in migrant MSM than in local MSM across all the age categories. However, all these differences were not statistically significant.

### Multivariate associations between HIV/STIs risks and migratory status

Table 3 presents the results of multivariate logistic regression of HIV/STIs risks on migratory status. After adjusting for socio-demographics, migrant MSM were more likely to self-report as heterosexual or uncertain about sexual orientation (OR = 1.5; 95% CI [1.0–2.4]) than local MSM. Condom use at homosexual debut was lower in migrant MSM than local MSM (OR = 0.7; 95% CI [0.5–0.9]). Migrant MSM have less odds of reporting HIV/STIs testing in the previous 6 months relative to local MSM (OR = 0.5; 95% CI [0.4–0.8]). In addition, migrant MSM indicated a lower level of HIV knowledge than local MSM (OR = 0.4; 95% CI [0.2–0.8]). Migrant MSM, compared to local MSM, report insignificant higher odds of UAI (OR = 1.4; 95% CI [0.9–2.0]) and having multiple homosexual partners (OR = 1.2; 95% CI [0.8–1.8]) in the past 6 months, but the confidence intervals indicate that suggest possible association if our sample size increases. We also did not find a significant association between migratory status and HIV/syphilis infection (OR = 1.9; 95% CI [0.9–4.0]).

**Table 3:** Multivariate logistic regression of HIV/STIs risks on migratory status, Guangzhou, China, 2010 (*N* = 522).

Characteristics	Overall		Adjusted OR (95% CI)^§^		*p* value
	*N*	%			
Self-reported sexual orientation^a^					0.072
Homosexual or bisexual	378	72.4	1.0	
Heterosexual or uncertain	144	27.6	1.5	1.0–2.4	
Condom use at homosexual debut^b^					0.032
No	255	48.9	1.0	
Yes	267	51.1	0.7	0.5–0.9	
UAI in the past 6 months					0.138
No	348	66.7	1.0	
Yes	174	33.3	1.4	0.9–2.0	
Multiple sexual partners in the past 6 months					0.284
No	238	45.5	1.0	
Yes	283	54.5	1.2	0.8–1.8	
Heterosexual vaginal sex in the previous 6 months					0.203
No	443	84.9	1.0	
Yes	79	15.1	1.5	0.8–2.6	
HIV/STIs testing in the past 6 months^c,¶^					0.003
No	280	53.6	1.0	
Yes	242	46.4	0.5	0.4–0.8	
Utilization of HIV/STIs prevention services in the past 6 months					0.144
No	195	37.4	1.0	
Yes	327	62.6	1.4	0.9–2.1	
HIV knowledge^d^					0.017
Not informed	43	8.2	1.0	
Informed	479	91.8	0.4	0.2–0.8	
HIV/syphilis infection^$^					0.091
Negative	485	92.9	1.0	
Positive	37	7.1	1.9	0.9–4.0	

**Notes.**

§ except for the characteristics presented in table, our model additionally adjusted for age, income and education Likelihood ratio test for interaction with age: a, Chi-square = 0.71 *p* = 0.3995; b, Chi-square = 2.15, *p* = 0.2419; c, Chi-square =0.29 *p* = 0.5928; d, Chi-square = 2.62 *p* = 0.1056. ¶, Combine HIV testing and other STIs testing; $, Combine HIV infection and syphilis infection.

Abbreviation UAI Unprotected anal intercourse STIs Sexually transmitted infections

## Discussion

Our study identified several noteworthy differences of HIV/STIs risks between migrant MSM and local MSM, which included UAI, having multiple homosexual partners, condom use at homosexual debut, HIV/STIs testing behavior and HIV knowledge. Because there is a paucity of studies comparing migrant MSM to local MSM, findings from our study will not only provide critical insights for understanding the role of internal migration in spreading HIV in MSM community in China but also contribute to intervention development targeting migrant MSM.

Our data suggest that migrant MSM, compared to local MSM, are at higher risk of having UAI and having multiple homosexual partners in the past 6 months. Our findings are similar to limited studies that compare migrant MSM with non-migrant MSM. For example, a study in Jinan, China indicated that migrant MSM were more likely to engage in UAI than non-migrant MSM (OR = 1.7; 95% CI [1.1–2.6]) (Ruan et al., 2008). Although our estimates were not statistically significant (e.g., UAI (OR = 1.4; 95% CI [0.9–2.0]), having multiple homosexual partners (OR = 1.2; 95% CI [0.8–1.8])), if we correctly interpret statistical testing and confidence intervals (Greenland et al., 2016), the confidence intervals clearly indicate that migration has a slight or moderate association with UAI and having multiple homosexual partners in the past 6 months. Previous studies in the United States hypothesized that the anonymity of living in a new city, often without social connections to home communities or the loss of close family relationships, might contribute to the increase of sexual risks in migrants (Bianchi et al., 2007; Egan et al., 2011). This finding underscores that migrant MSM, compared to local MSM, were more likely to have sexual risk behaviors, which calls for a targeted intervention approach to reduce sexual risk behaviors among migrant MSM.

Our study identified differences of HIV/STIs testing between migrant MSM and local MSM. Generally, due to China’ s household registration system and urban social security system, migrants have limited access to comprehensive, convenient and long-term health services (Peng et al., 2010). Previous research has further indicated that migrants, compared to local residents, have less access to HIV testing. For example, a recent study, conducted in migrant women and local women, suggested that HIV testing was less common in migrant women than in local women (Song et al., 2015). Our results were similar with this study by indicating migrant MSM also have less access to HIV/STIs testing compared with local MSM. HIV testing is crucial and fundamental component of HIV prevention. HIV testing and diagnosis can lead to the initiation of antiretroviral treatment and can promote behavioral change, both of which can optimize clinical outcomes and reduce the risk of HIV transmission to others (National Center for HIV/AIDS, Viral Hepatitis, STD and TB Prevention, 2011). Findings from our study underscore the specific need to promote HIV/STIs testing among the migrant MSM population.

Elevated prevalence of HIV and other STIs infections in migrants was consistently reported in international and domestic studies (Egan et al., 2011; Evans et al., 2011; He et al., 2006; Ruan et al., 2009; Wang et al., 2010). Although our study failed to identify a significant association between migratory status and HIV/syphilis infection, the confidence interval of OR (OR = 1.9; 95% CI [0.9–4.0]) implies that there might be an association if the sample size increases. Further large sample size studies are warranted to test our hypothesis. Our study also suggests that migrant MSM reported a lower level of HIV knowledge compared with local MSM. Previous studies have suggested that the lack of HIV knowledge is a risk factor of HIV/STIs infection in migrant MSM (Wang et al., 2013; Wong et al., 2008). Since the early 21st century, large-scale interventions have been conducted to tackle the dramatic increase of sexually transmitted HIV in China, however no wide-scale intervention targeting migrants was conducted (Rou et al., 2010). Furthermore, migrants are often neglected by the general health system in the host cities, which further reduces their opportunities to receive regular HIV prevention services, especially the HIV prevention knowledge. Our findings urge the specific intervention programs targeting migrant MSM or the integration of migrants’ HIV prevention services to the general health system in the host cities where migrants live.

Several limitations have to be recognized before interpreting our results. First, since our study was a cross-sectional study, temporal relations could not be assessed so that we cannot make any casual inference. The second limitation pertained to the sampling procedure; convenience sampling undermines our generalizability of findings. Finally, information bias is highly possible given self-reported data and relatively sensitive questions we asked.

## Conclusion

Homosexual transmission of HIV is increasingly playing a significant role in China’s HIV epidemic. However, very few studies have been conducted to understand the associations between migratory status and HIV risks in the MSM population. Findings derived from this study contribute to better understanding of the differences of HIV/STIs risks between migrant MSM and local MSM. Furthermore, our study reveals that migrant MSM are at higher risk of contracting HIV and other STDs due to their higher odds of engaging in sexual risk behaviors, lower HIV knowledge level and less access to HIV testing. These findings have significant implications for China’s HIV prevention efforts. First, given migrant MSM reported higher level sexual risk behaviors than local migrant, behavioral interventions, such as condom promotion, peer education, etc., need to be enhanced in the migrant MSM population. Second, the significant lower level of HIV knowledge observed in migrant MSM urges the health professionals to improve the knowledge dissemination, either its content or its delivery, to make it more absorbable or accessible to migrant MSM. Third, a relatively lower HIV testing uptakes in migrant MSM may suggest possible structural barriers to having HIV test (Song et al., 2011), free and easily accessible HIV testing needs to be provided in migrant MSM.

##  Supplemental Information

10.7717/peerj.2169/supp-1 Supplemental Information 1 Example Stata Codes

10.7717/peerj.2169/supp-2 Supplemental Information 2 Raw data

## Additional Information and Declarations

### Data Availability

The following information was supplied regarding data availability: The raw data has been supplied as Supplemental Information 2.
