# Supplementary material for: HIV/STIs risks between migrant MSM and local MSM: a cross-sectional comparison study in China
Source: PeerJ. 2016 Jul 13;4:e2169. doi: 10.7717/peerj.2169 (PMC4950534; doi:10.7717/peerj.2169)
Supplement: Supplemental Information 1 [file peerj-04-2169-s001.docx]

#### Descriptive analysis example###

### t test for continuous variables###

ttest age, by(migration)

### chi-square or exact test for categorical variables###

tab marriage migration, chi2 exact

###logistic regression for our study####

logistic migration orientation ib2.firstsex_condom ib0.UAI mul_partner femalesex hiv_sti_testing service_access knowledge_group hiv_syphilis age income edu

####likelihood ratio test for interaction term###

lrtest model1 model2

#model1 is the model with interaction term, model2 is the model without interaction term#

####likelihood ratio test for interaction term###

graph bar (mean) hiv_syphilis, over(migration) over(age_group)
